# American Medical Association

**Published:** 1881-04-20

**Authors:** 


### EDITORIALS AND MISCELLANEOUS.

### *THE AMERICAN MEDICAL ASSOCIATION.*

This Association meets in Richmond, Virginia, on May 3d, 1881. A full turn-out and a pleasant time is expected. The Senior Editor of this Journal contemplates being present, and anticipates much pleasure and profitable instruction in meeting with the brethren of the profession from all sections of the Union. The following are the delegates appointed to that body by the Georgia Medical Association, to-wit: Drs. Henry Gaither, Thos. S. Powell, H. F. Scott, Thos. R Wright, J. Thad Johnson, Henry F. Campbell, Wm. F. Holt, J. W. Alfriend, Robert Battey, W. J. Harrell, T. M. McIntosh, J. C. LeHardy, F. A. Sanford, W. B. Wells, A. L. Campbell, K. P. Moore, C. H. Hall, Geo. F. Cooper, W. F. Westmoreland, A. W. Griggs, DeSaussure Ford, Jno. G. Thomas, J. W. Bailey, Thos. Raines, A. G. Whitehead, J. B. Holmes, J. P. Logan, A. C. Goodrich, Chas. W. Hickman, Jno. D. Martin, W. O. Daniel.

